# Association between gut microbiota and anxiety disorders: a bidirectional two-sample mendelian randomization study

**DOI:** 10.1186/s12888-024-05824-x

**Published:** 2024-05-27

**Authors:** Jianbing Li, Changhe Fan, Jiaqi Wang, Bulang Tang, Jiafan Cao, Xianzhe Hu, Xuan Zhao, Caiqin Feng

**Affiliations:** 1 Department of Psychiatry, Guangdong Second Provincial General Hospital, Guangzhou, 510317 PR China; 2 School of Pharmacy, Guangdong Pharmaceutical University, Guangzhou, 510006 China

**Keywords:** Anxiety disorders, Gut microbiota, Mendelian randomization, Single nucleotide polymorphism

## Abstract

**Background:**

There are many articles reporting that the component of intestinal microbiota implies a link to anxiety disorders (AD), and the brain-gut axis is also a hot topic in current research. However, the specific relevance between gut microbiota and AD is uncertain. We aimed to investigate causal relationship between gut microbiota and AD by using bidirectional Mendelian randomization (MR).

**Methods:**

Genetic instrumental variable (IV) for the gut microbiota were obtained from a genome-wide association study (GWAS) involving 18,340 participants. Summary data for AD were derived from the GWAS and included 158,565 cases and 300,995 controls. We applied the inverse variance weighted (IVW) method as the main analysis. Cochran’s Q values was computed to evaluate the heterogeneity among IVs. Sensitivity analyses including intercept of MR-Egger method and MR-PRESSO analysis were used to test the horizontal pleiotropy.

**Result:**

We discovered 9 potential connections between bacterial traits on genus level and AD. Utilizing the IVW method, we identified 5 bacterial genera that exhibited a direct correlation with the risk of AD: genus *Eubacteriumbrachygroup*, genus *Coprococcus3*, genus *Enterorhabdus*, genus *Oxalobacter*, genus *Ruminiclostridium6*. Additionally, we found 4 bacterial genera that exhibited a negative association with AD: genus *Blautia*, genus *Butyricicoccus*, genus *Erysipelotrichaceae-UCG003* and genus *Parasutterella*. The associations were confirmed by the sensitivity analyses.

**Conclusion:**

Our study found a causal relation between parts of the gut microbiota and AD. Further randomized controlled trials are crucial to elucidate the positive effects of probiotics on AD and their particular protection systems.

**Supplementary Information:**

The online version contains supplementary material available at 10.1186/s12888-024-05824-x.

## Introduction

Anxiety disorders (AD), being the prevailing mental disorders, have a substantial impact on individuals and society alike [1]. The core features of AD contain indiscriminate anxiety and fear or elusion of persistent and debilitating threats, resulting in substantial medical costs and a burdensome morbidity burden [1, 2]. As one of the most popular mental illnesses among young individuals, AD are also the earliest-onset mental disorders [3]. Amidst the COVID-19 pandemic, there has been a significant surge in the occurrence of AD among children, adolescents, and young adults globally [4]. First-line treatments for AD include medication and psychotherapy [5]. However, medication treatments carry certain side effects and risks, such as dependence, cognitive impairment, and an increased risk of heart disease [6]. The majority of individuals suffering from AD lack access to efficacious treatment options, leaving them vulnerable to relapse [7, 8].

Many studies have shown that the occurrence of AD is related to changes in intestinal flora [9, 10]. In social anxiety disorder (SAD), there was an increase in the relative abundance of *Anaeromassillibacillus* and *Gordonibacter* genera, whereas healthy controls exhibited an enrichment of *Parasuterella* [11]. Another article found a reduction in *Eubacterium rectale* and *Fecalibacterium*, as well as an increase in *Escherichia*, *Shigella*, *Fusobacterium*, and *Ruminococcus* in patients with generalized anxiety disorder (GAD) [12]. In addition, there are numerous documents demonstrating an association between the gut microbiota and mental illness, and the modulation of the gut microbiota on the gut-brain axis has garnered significant attention, such as an elevation of *Enterobacteriaceae* and *Desulfovibrio*, and a reduction of *Faecalibacterium* in patients with AD [10, 13–17]. In the aforementioned section, it was observed that the evidence exhibits complexities and disparities, as well as some contradictory results, potentially stemming from various confounding factors among different studies.

The previous studies examining the connection between gut microbiota and AD have predominantly relied on cross-sectional designs, which limits the ability to establish a causal relationship between these associations. Therefore, unraveling the causal mechanisms behind gut microbiota-derived AD not only enhances our understanding of their pathogenesis but also provides valuable guidance for implementing microbiota-directed interventions in clinical settings to address AD. Previous Mendelian randomization (MR) studies have primarily focused on investigating the causal relationship between oral microbiota abundance and AD, or between gut microbiota and other psychiatric disorders. A systematic MR study specifically examining the causal relationship between gut microbiota and AD is still lacking in the current literature. In light of this, it is imperative to unravel the causal link between the gut microbiota and AD.

MR is a statistical approach that infers a causal relationship with exposure to a result. It leverages genetic variations linked to the exposure as a proxy for the exposure itself, enabling the assessment of the association between the exposure and the outcome [18]. Due to the highly effective findings of large-scale genome-wide association study (GWAS) at the gut microbiota and disease level, MR analysis has been abroad used in many scenarios, such as between the oral microbiome and AD, relations between genetically determined metabolites and anxiety symptoms [19, 20]. However, there are no specific studies on the causal relationship between gut microbiota and AD. In this research, we applied a bidirectional two-sample MR method to investigate causal relationship between the gut microbiota and AD.

## Materials and methods

### The assumptions and study design of MR

**Fig. 1:**
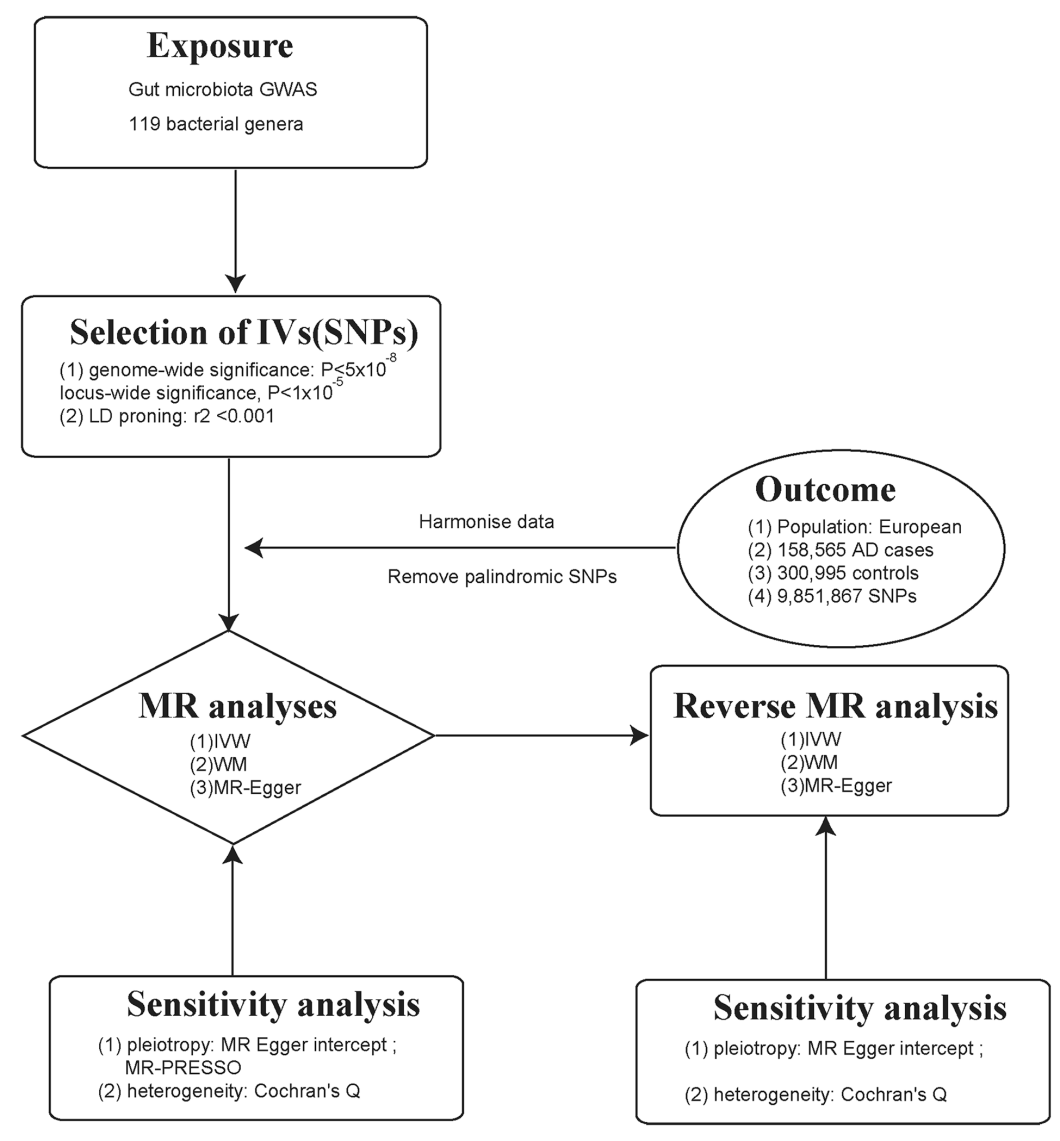
A flowchart illustrating the MR analysis process for the association between gut microbiota and AD

MR is a methodology employed to assess causal associations between variables. In order to ensure the validity of MR analysis, 3 fundamental assumptions must be met: (i) the instrumental variable (IV) exhibits a strong link to the exposure factor, (ii) the IV remains unaffected by potential confounding factors., and (iii) the IV influences the result factor solely via the exposure factor [21]. By applying strict selection criteria, appropriate SNPs were selected as IV for conducting MR analysis on two independent samples. The main aim was to examine the causal relationship between gut microbiota and AD. Furthermore, this study adhered to the guidelines outlined in the Strengthening the Reporting of Observational Studies in Epidemiology-Mendelian Randomization (STROBE-MR) framework [22] (Fig. 1).

### Data sources

The data on gut microbiota GWAS used in this study were obtained from an overall meta-analysis conducted by the MiBioGen consortium. The meta-analysis comprised a total of 18,340 individuals from 24 different groups. The alliance combines human whole-genome genotyping with fecal 16 S rRNA sequencing data to perform thorough research and analysis. The large-scale, multi-ethnic genome-wide meta-analysis provided valuable insights into the genetic influences on the gut microbiome composition [23]. The GWAS data on the gut microbiome can be integrated into MR studies to explore the causal relationship between genetic variations in the gut microbiome and phenotypic traits, providing valuable insights into the role of the microbiome in human health and disease.

As for the data on genetic variants linked to AD, they were sourced from the Medical Research Council Integrative Epidemiology Unit (MRC-IEU) consortium. The cases were defined as individuals who had sought medical attention for symptoms of nervousness, anxiety, or depression. The study population consisted of individuals of European descent, comprising both males and females, and the data were sourced from the year 2018. The dataset included a total of 158,565 cases and 300,995 controls. The diagnosis was based on self-report questionnaires. Detailed information regarding the data origins for this MR study can be found in Table 1 [24, 25].

**Table 1:** Information regarding the GWAS and datasets employed in our analyses is provided in detail

Exposure or outcome	Sample size	Ancestry	Links for data download	PMID
Human gut microbiome	18,340 participants	Mixed	https://mibiogen.gcc.rug.nl	33462485
Anxiety	158,565 cases; 300,995 controls	European	Trait: Seen doctor (GP) for nerves, anxiety, tension or depression - IEU OpenGWAS project (mrcieu.ac.uk)	

### Selection of IV

The GWAS data of exposure contained a total of 5 taxonomic levels for 211 bacterial groups. The genus level is the smallest and most specific classification level. To accurately identify each pathogenic bacterial group, we focused our analysis only on the genus level, specifically examining 131 bacterial classifications. After excluding 12 unknown groups, a total of 119 bacterial genera were included in the study.

To fulfill the demands of MR studies, our initial step involved the SNPs that exhibited an intense association with the exposure factors. However, when employing a stringent threshold of (*P* < 5 × 10^− 8^), we obtained a limited number of IVs. Consequently, we adjusted the threshold to (*P* < 1 × 10^− 5^) to ensure the inclusion of more IVs, thereby enabling robust and reliable results. For the selection of IVs associated with AD in the reverse MR analysis, a heightened level of stringency was implemented by applying a *P*-value threshold of *P* < 5 × 10^− 8^.

**Fig. 2:**
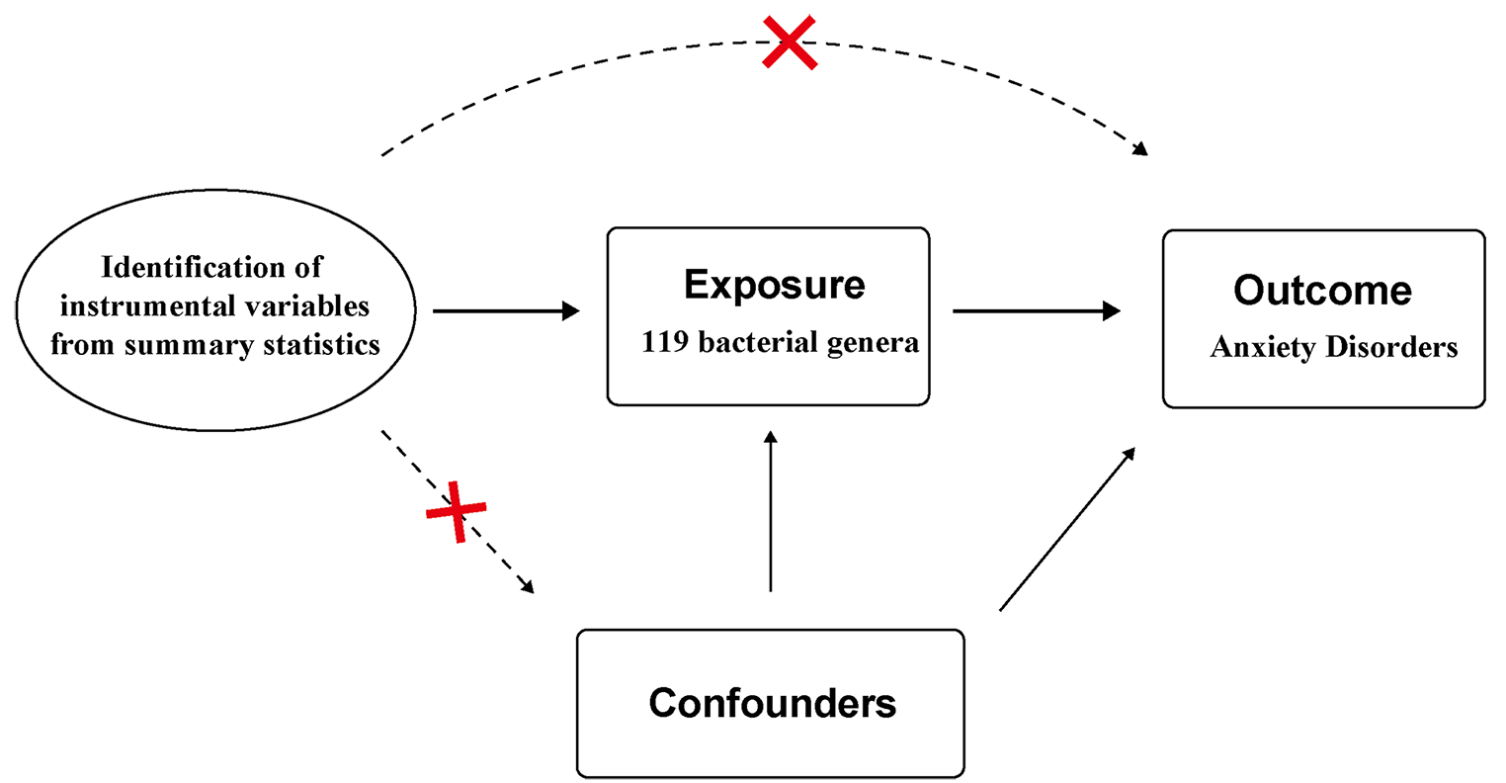
Assumptions in MR studies: a brief overview

We utilized the F-statistic to further evaluate the instrument strength. The F-statistic was determined using the formula: F = *β*^2^ / SE^2^. This statistic provided an assessment of the overall instrument strength [26] (Fig. 2). An F-statistic exceeding 10 was considered indicative of an intense conjunction between the IV and the exposure. Besides *P*-value threshold, the F statistic in our analysis would provide additional information on the instrument strength beyond *P*-value.

### Statistical analysis

The primary methodology employed in MR analysis is the inverse variance weighting (IVW) method. This approach utilizes a meta-analysis technique to combine the Wald estimates connected to individual single nucleotide polymorphisms (SNPs), providing comprehensive estimate of the collective impact of gut microbiota on AD. A crucial assumption in MR is the absence of horizontal pleiotropy, where the IV has a direct impact on the outcome variable solely through the exposure factor, without any influence from through alternative pathways. When this assumption is satisfied, the IVW method can provide estimates that are consistent and estimates [27]. In cases where a causal relationship (*P* < 0.05) is established by the IVW method, two alternative approaches, namely MR-Egger and the weighted median approach, are utilized to supplement an enrich the IVW results. The MR-Egger method relaxes the assumption of a zero intercept, and it can estimate causal effects, even pleiotropy was presented in IVs. The intercept in the MR-Egger method can indicate the extent of horizontal pleiotropy [27]. These additional methods provide valuable insights and strengthen the overall analysis by considering potential biases and alternative causal pathways.

The weighted median method can return unbiased causal estimate when only 50% of SNPs are valid [28]. In this study, we employed a significance threshold of *P* < 0.05 to determine statistical significance, and the assessment of causality was expressed through odds ratios (OR) and 95% confidence intervals (CI). In instances where causal relationships were established, unidentified taxa were excluded, and additional sensitivity analyses were performed to guarantee the stability of the consequences. The false discovery rate (FDR) is utilized to control for multiple testing and reduce the likelihood of false positive findings. All of the aforementioned analyses were performed utilizing the TwoSampleMR package (version 0.5.7) in R (version 4.3.0), providing a robust and standardized approach to MR analysis.

## Results

**Fig. 3:**
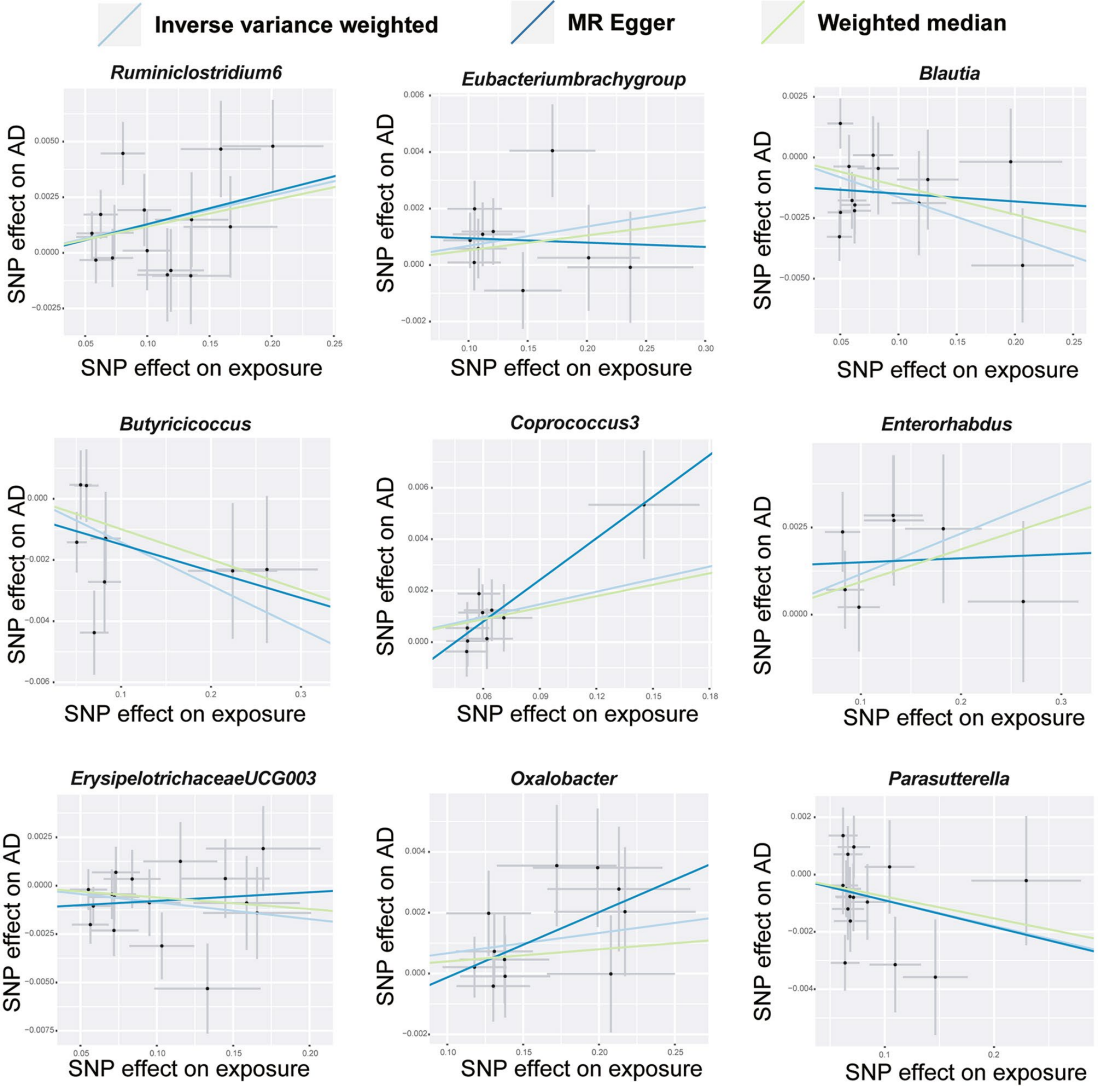
The scatter plots depict the causal relationship between gut microbiota and AD

According to the criteria for IV selection, a total of 1,531 SNPs were identified and selected as IV associated with gut microbiota. The F-statistics for these IVs all exceed 10, suggesting that the estimated coefficients are improbable to be influenced by the bias caused by weak instruments. Supplementary Tables 1 and 2 provides detailed information about the selected IVs. None of the SNPs were involved in more than one of the association results in Fig. 3.

The majority of gut microbiota showed no significant correlation with AD. However, using the IVW method, we identified 9 bacterial features that were significantly associated with the risk of AD on genus level (Supplementary Table 3). We used 3 methods, IVW, weighted median and MR-Egger, and defined *P* < 0.05 for IVW method screening as a positive result.

**Fig. 4:**
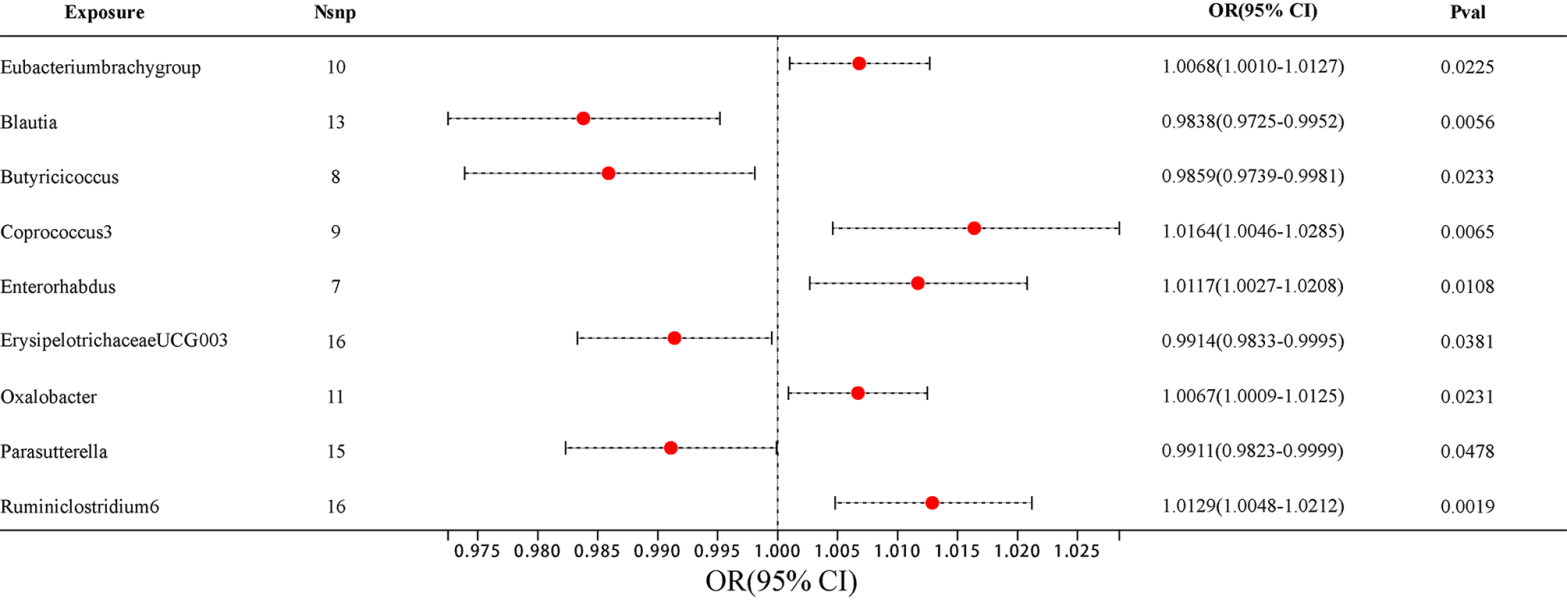
The forest plot illustrates the connections between 9 bacterial genus traits and the likelihood of developing AD

Among them, 4 bacterial genera are negatively correlated with AD, indicating that a higher genetically predicted a lower risk of for AD (Fig. 4 and Supplementary Table 4). They are: genus *Blautia* (OR = 0.9838, 95% CI, 0.9725–0.9952, *P* = 0.0056), genus *Butyricicoccus* (OR = 0.9859, 95% CI, 0.9739–0.9981, *P* = 0.0233), genus *ErysipelotrichaceaeUCG003* (OR = 0.9914, 95% CI, 0.9833–0.9995, *P* = 0.0381) and genus *Parasutterella* (OR = 0.9911, 95% CI, 0.9823–0.9999, *P* = 0.0478). Supplementary Table 4 shows the completed data. In sensitivity analysis, MR-Egger, weighted median demonstrated consistent results, except for genus *ErysipelotrichaceaeUCG003*, where the MR-Egger trend was in the contrary direction compared to IVW and weighted median.

Another 5 bacterial genera showed a positive correlation with AD, genus *Eubacteriumbrachygroup* (OR = 1.0068, 95% CI, 1.0010–1.0127, *P* = 0.0225), genus *Coprococcus3* (OR = 1.0164, 95% CI, 1.0046–1.0285, *P* = 0.0065), genus *Enterorhabdus* (OR = 1.0117, 95% CI, 1.0027–1.0208, *P* = 0.0108), genus *Oxalobacter* (OR = 1.0067, 95% CI, 1.0009–1.0125, *P* = 0.0231) and genus *Ruminiclostridium6* (OR = 1.0129, 95% CI, 1.0048–1.0212, *P* = 0.0019) (Fig. 4 and Supplementary Table 4). In the MR-Egger method, the trends of genus *Eubacteriumbrachygroup* are different from those of the IVW and WM methods.

In horizontal pleiotropy analysis, we used the MR-Egger method and found *P*-value of the MR-intercept were all greater than 0.05. In addition, further MR PRESSO analysis was conducted, ruling out the existence of horizontal pleiotropy (*P* > 0.05) (Supplementary Tables 5 and 6). To assess the heterogeneity of gut microbiome IVs, we employed Cochran’s Q test statistics, which revealed no heterogeneity among the gut microbiome IVs (*P* > 0.05) (Supplementary Table 7).

Reverse MR analyses were conducted to examine the links between the 9 bacterial genera and AD. No significant statistical relationship was observed using the IVW method: genus *Eubacteriumbrachygroup* (OR = 1.4058, 95% CI, 0.4060–4.8674, *P* = 0.5909), genus *Blautia* (OR = 0.9453, 95% CI, 0.5572–1.6038, *P* = 0.8348), genus *Butyricicoccus* (OR = 0.9834, 95% CI, 0.5704–1.6952, *P* = 0.9518), genus *Coprococcus3* (OR = 0.8886, 95% CI, 0.5040–1.5667, *P* = 0.6831), genus *Enterorhabdus* (OR = 1.0383, 95% CI, 0.4168–2.5868, *P* = 0.9356), genus *ErysipelotrichaceaeUCG003* (OR = 0.6593, 95% CI, 0.3556–1.2221, *P* = 0.1858), genus *Oxalobacter* (OR = 1.2849, 95% CI, 0.4021–4.1051, *P* = 0.6724), genus *Parasutterella* (OR = 0.7245, 95% CI, 0.3713–1.4136, *P* = 0.3447), genus *Ruminiclostridium6* (OR = 0.7095, 95% CI, 0.3825–1.3162, *P* = 0.2764) (Supplementary Tables 8 and 9).

## Discussion

In the context of this study, we used two-sample MR studies to discover the link between AD and gut microbiota. Among the 9 bacterial genus we found, 4 bacteria were negatively correlated with AD and may have a positive effect on AD, and the other 5 bacteria were positively correlated with the occurrence of AD and may promote the development of AD.

*Blautia stercoris MRx0006* has been shown to alleviate social dysfunctions, monotonous behaviors, and anxiety-like behaviors relevant to autism disorders in a mouse model. MRx0006 administration at the microbial level, as observed by Paromita Sen et al., resulted in a reduction in the abundance of Alistipes putredinis, which likely underlie the observed increase in expressions of oxytocin, arginine vasopressin, and their receptors, ultimately leading to improved behavioral outcomes [29]. *Butyricicoccus* was also inversely associated with AD in a cross-sectional study, which is consistent with our findings [12]. Approximately 70% of individuals with autism spectrum disorder (ASD) exhibit comorbid symptoms of anxiety, and the findings from a published article confirming the decreased relative abundance of *ErysipelotrichaceaeUCG003* in ASD patients further support our research results indicating a negative correlation between *ErysipelotrichaceaeUCG003* and AD [30]. In a study examining SAD, the control group exhibited higher levels of the positive bacteria *Parasutterella* compared to the anxiety group. The term “psychobiotics” has been coined to refer to these microbes that are associated with improved mood [11]. However, in a study by Yi Zhang et al., a psychological stress model was established in C57BL/6J mice, followed by fecal microbiota transplantation using samples from stressed (S) and non-stressed (NS) mice. The results showed an increased abundance of *Parasutterella* in S mice and mechanistic analysis suggested its potential involvement in negative regulation of metabolism. Despite this controversial finding, our study utilized MR to reveal a negative association between *Parasutterella* and anxiety disorders. However, further experimental investigations are required to elucidate the underlying molecular mechanisms [31].

Five bacterial genera positively linked to anxiety may indicate that they exacerbate anxiety, but they were less reported. In a study in which consuming prebiotics altered the microbiota of healthy adults, the prebiotics reduced *Eubacteriumbrachygroup* but did not significantly change biomarkers of stress or mental health symptoms [32]. In previous studies on AD cases, it has been found that individuals with AD have lower levels of *Coprococcus* [33]. However, in our study, we observed an increasing trend in *Coprococcus3*, despite belonging to the same genus. This suggests that even within the same genus, the impact of different genus may vary. In contrast to our findings, *Enterorhabdus* exhibited a declining pattern in a mouse model of anxiety and depression induced by social defeat [34]. This observation highlights the influence of various factors on alterations in gut microbiota, which may diverge across different species.

Nevertheless, it is crucial to acknowledge that our study has certain limitations. First, the results of this analysis are limited to European populations and may not be generalizable to other populations. Secondly, we observed that the adjusted *P*-values remained relatively large after multiple test adjustment. The reduced statistical power resulting from the limited sample size may also constrain our ability to detect significant associations between variables. Finally, proving the direct impact of sample types on the outcomes is challenging. However, the selection of sample types is often constrained by the availability of suitable genetic instruments and relevant data sources. The dataset we utilized does not provide specific information on the dietary habits of the individuals or their other medical conditions. Therefore, further examination and validation are needed in the future.

## Conclusion

In summary, utilizing large-scale GWAS analysis, MR studies have disclosed a causal relationship between gut microbiota and AD. Among these, 4 bacterial genera exhibited a negative correlation, while 5 bacteria genera showed a positive correlation with AD. However, further exploration of the mechanisms linking gut microbiota to AD requires the establishment of larger GWAS databases. Several gut bacteria have been identified to reduce the occurrence of anxiety, offering promising prospects for the treatment and precaution of AD. Subsequent research should prioritize the exploration of the underlying mechanisms and the development of targeted interventions based on these findings.

### Electronic supplementary material

Below is the link to the electronic supplementary material.

Supplementary Material 1

Supplementary Material 2

Supplementary Material 3

Supplementary Material 4

Supplementary Material 5

Supplementary Material 6

Supplementary Material 7

Supplementary Material 8

Supplementary Material 9

## Data Availability

The raw data analyzed during the current study were available in public databases including IEU database(ukb-b-6991) and MiBioGen database(https://mibiogen.gcc.rug.nl). The code and data related to this study are available from the corresponding author upon reasonable request.

## Abbreviations

AD: Anxiety disorders
MR: Mendelian randomization
IV(s): Instrumental variable(s)
GWAS: Genome-wide association study
MRC-IEU: Medical Research Council Integrative Epidemiology Unit
IVW: Inverse variance weighting
SAD: Social anxiety disorder
GAD: Generalized anxiety disorder
STROBE-MR: Strengthening the Reporting of Observational Studies in Epidemiology-Mendelian Randomization
SNP(s): Single nucleotide polymorphism(s)
OR: Odds ratios
CI: Confidence intervals
ASD: Autism spectrum disorder
MDD: Major depressive disorder
